# Coupling and Uncoupling Pleiotropy Between Hypertension and Type 2 Diabetes Contribute to Exploring Potential Heterogeneity in Cardiovascular Risk in East Asian Population

**DOI:** 10.3390/biomedicines14061404

**Published:** 2026-06-22

**Authors:** Huan Yu, Liuyan Zheng, Jingxian Wu, Ruotong Yang, Kun Wang, Huairong Wang, Shuting Xie, Yalin Chen, Teng Li, Xueying Qin, Yonghua Hu, Yiqun Wu

**Affiliations:** 1 Department of Epidemiology and Biostatistics, School of Public Health, Peking University Health Science Center, 38 Xueyuan Road, Beijing 100191, China; yuhh@pku.edu.cn (H.Y.); zly101759@163.com (L.Z.); yangruotong@bjmu.edu.cn (R.Y.); 1810306239@pku.edu.cn (K.W.); yhhu@bjmu.edu.cn (Y.H.); 2 Key Laboratory of Epidemiology of Major Diseases (Peking University), Ministry of Education, Beijing 100191, China; 3 Department of Medical Epidemiology and Biostatistics, Karolinska Institute, 171 65 Stockholm, Sweden

**Keywords:** comorbidity, type 2 diabetes, hypertension, pleiotropy, cardiovascular disease

## Abstract

**Background/Objectives**: This study aims to systematically deconstruct the shared genetic architecture underlying the comorbidity of hypertension (HTN) and type 2 diabetes (T2D) and evaluate how these divergent genetic architectures are associated with differential cardiovascular risk in East Asian population. **Methods**: This two-stage study first leveraged the largest genetic dataset from >300,000 East Asian individuals to identify pleiotropic loci between hypertension and type 2 diabetes using conjunctional false discovery rates, classifying them into coupling and uncoupling types based on effect directions. Corresponding polygenic risk scores (PRSs) were then constructed and validated in an independent family-based cohort. A logistic regression model was used to examine the associations between different genetic architectures and cardiovascular risk, including comorbidity onset and cardiovascular outcomes. **Results**: A total of 463 pleiotropic loci were identified, including 439 coupling loci and 24 uncoupling loci. The coupling PRS showed a significant association with single T2D (OR = 1.53; 95% CI: 1.08–2.18; *p* = 0.017), whereas the other associations were not significant, although the effect estimates were directionally consistent with our hypothesis. Crucially, coupling and uncoupling PRSs showed divergent cardiovascular risk profiles and exhibited distinct gene–environment interactions. **Conclusions**: Our findings suggest that coupling and uncoupling pleiotropy between hypertension and type 2 diabetes may contribute to the heterogeneity of cardiovascular risk in East Asians. Deconstructing genetic pleiotropy offers a potential framework for precision prevention strategies, although these findings are exploratory and warrant further validation in larger cohorts.

## 1. Introduction

Hypertension (HTN) and type 2 diabetes (T2D) are the two most prevalent chronic metabolic disorders worldwide, posing a formidable challenge to global public health [1,2,3,4,5,6,7,8]. Moreover, they frequently co-occur as comorbidities [9,10,11], and this comorbid state is not merely an additive combination of individual risks; instead, through synergistic interactions, it multiplies the risk of complications, such as coronary heart disease, stroke, and diabetic nephropathy, far beyond that associated with either condition alone [12,13,14,15,16]. Most current studies have focused on HTN and T2D in isolation [17,18,19,20], limiting their ability to explore the pleiotropy observed between them, although recent studies revealed an estimated genetic correlation exceeding 40% [20,21].

Genetic pleiotropy is central to unraveling the biological basis of comorbidity among complex diseases. However, growing evidence suggests that pleiotropy is not uniform but rather categorizable, and that different types of pleiotropy may exert distinct effects on disease risk [22,23,24]. Specifically, it may be broadly classified into two distinct forms: coupling pleiotropy, in which a single allele exerts concordant effects—either increasing or decreasing risk—on both conditions; and uncoupling pleiotropy, a concept akin to antagonistic pleiotropy [25], in which the same allele has opposing effects on the two diseases [22]. Theoretically, these distinct architectures imply divergent clinical trajectories, where coupling pleiotropy is hypothesized to be associated with a higher burden of comorbidity and more severe cardiovascular outcomes, while uncoupling pleiotropy may relate to more disease-specific manifestations or trade-offs. Despite this conceptual advance, the roles of these two pleiotropic architectures in the comorbidity of HTN and T2D remain poorly understood. It remains unclear whether there exist specific genetic variants that exert distinct or even opposing functional impacts on hypertension versus T2D and consequently drive divergent long-term cardiovascular outcomes. Key unanswered questions include how they shape the onset and trajectory of comorbidity, whether they drive divergent cardiovascular disease risks, and whether they interact with one another or with environmental and clinical factors. Moreover, the vast majority of existing pleiotropy studies have been conducted in European ancestry populations, leaving a critical gap in evidence regarding East Asian populations.

Therefore, we designed a two-stage study, which leveraged data from two independent cohorts, utilizing the largest currently available genetic dataset of East Asian ancestry, to explore heterogeneity between coupling and uncoupling pleiotropic effects of HTN-T2D comorbidity and their impact on cardiovascular risk. This study will provide novel insights into the distinct roles of genetic pleiotropy between HTN and T2D and offer a scientific foundation for precision management and risk stratification in cardiovascular disease prevention based on coupling and uncoupling forms of pleiotropy in East Asian population.

## 2. Materials and Methods

### 2.1. Data Resources

The data used in this study came from the Taiwan Precision Medicine Initiative (TPMI) and the Fangshan Family-based Ischemic Stroke Study in China (FISSIC). The TPMI is a large-scale multi-center cohort in Taiwan [26,27]. As of 28 December 2023, the TPMI has enrolled 565,390 participants of predominantly Han Chinese ancestry, who provided informed consent for genomic analysis and linkage to their electronic medical records, constituting the largest East Asian genetic cohort with available data to date. For our study, genome-wide association study (GWAS) summary statistics for HTN (85,661 cases and 248,898 controls) and T2D (59,289 cases and 269,125 controls) were generated from this cohort using standard protocols that account for the cohort’s population structure and familial relatedness, as detailed in the main TPMI publication [26,27]. The FISSIC is a community-based genetic epidemiological family cohort established in the rural Fangshan District, Beijing, China. The protocol of the study has been described previously [28]. The study was approved by the Ethics Committee of the Peking University Health Science Center (approval number: IRB00001052-13027), and all participants provided written informed consent. A total of 1205 individuals with available GWAS data were included in the final analysis. Characterized by low population mobility, minimal confounding due to population stratification, and a high prevalence of cardiovascular disease, the FISSIC cohort offers a valuable resource for genetic and epidemiological research.

### 2.2. Identification and Classification of Pleiotropic Loci

To systematically identify genetic variants influencing both HTN and T2D, we applied the conjunctional false discovery rate (cFDR) [29] framework to the TPMI GWAS summary statistics. This method leverages the cross-trait association signal to boost power for detecting pleiotropic loci beyond conventional GWAS significance thresholds [29,30,31]. We considered a variant as a significant pleiotropic locus if its cFDR value was < 0.05 [29].

Pleiotropic loci were then classified into coupling loci and uncoupling loci based on the direction of their allelic effects. Coupling loci were defined as variants where the same effect allele was associated with increased (or decreased) risk for both HTN and T2D—where beta coefficients had the same sign; and uncoupling loci were defined as variants where the same effect allele was associated with increased risk for one disease but decreased risk for the other—where beta coefficients had opposing signs [22].

### 2.3. Genetic Correlation Analysis and Colocalization Analysis

To quantify the overall shared genetic architecture between HTN and T2D, we estimated their genome-wide genetic correlation (rg) using MTAG [32] based on the TPMI cohort. To investigate whether the observed genetic correlation at specific genomic loci was driven by shared causal variants, we conducted colocalization analysis using the COLOC method. We considered a posterior probability for hypothesis H_4_ (PPH_4_) ≥ 0.8 as strong evidence of colocalization, indicating that the same underlying causal variant likely influences both HTN and T2D at that locus [33,34]. Details of MTAG and COLOC methods could be found in Method S1 Supplementary File S1.

### 2.4. Construction of Coupling/Uncoupling Polygenic Risk Scores

Polygenic risk scores (PRSs) were constructed to quantify individual genetic burden associated with the two distinct modes of pleiotropy between HTN and T2D using GWAS summary data from TPMI [26,27]. A total of 463 pleiotropic SNPs with a cFDR value < 0.05 were considered in this step. First, to ensure independence among pleiotropic SNPs and avoid LD-induced bias, we performed LD-based clumping on this set of lead SNPs using PLINK v2.0. Clumping was conducted with an r^2^ threshold of 0.1 within a 250 kb window [31], using a reference panel derived from Han Chinese ancestry target population. Second, lead SNPs from all identified pleiotropic loci were classified into coupling or uncoupling groups based on the concordance of their effect size signs in the TPMI-derived HTN and T2D GWAS, and further stratified into three biologically meaningful categories: (1) coupling—variants where the same allele increases or decreasing risk for both HTN and T2D; (2) T2D-risk-increasing uncoupling—variants where the allele increases T2D risk but decreases HTN risk; and (3) HTN-risk-increasing uncoupling—variants where the allele increases HTN risk but decreases T2D risk.

For each individual in FISSIC, a separate PRS was calculated for each of the three categories as the weighted sum of trait-increasing alleles:

$$PRS=\sum_{i=1}^{n}\omega_{i}\times {SNP}_{i}$$

where ${SNP}_{i}$ is the individual’s allele count for the effect allele. To tailor the PRS construction to the specific biological hypotheses of concordant and discordant pleiotropy, we employed a customized weighting strategy ($\omega_{i}$) rather than standard GWAS beta coefficients. This approach was designed to quantify the magnitude of shared genetic effects specific to each mode. Specially, weight $\omega_{i}$ was defined as the sum of the absolute beta coefficients from the HTN and T2D GWAS for coupling loci (to reflect synergistic risk) and as the absolute difference between the two betas for uncoupling loci (to capture discordant effects).

$$\omega_{i}=\left\{\begin{array}{c} {\beta_{i}^{HTN}+\beta_{i}^{T2D}}_{,for\;coupling\;SNPs\;(concordant\;effects)}\\ {\left|\beta_{i}^{HTN}-\beta_{i}^{T2D}\right|}^{,for\;uncoupling\;SNPs\;(discordant\;effects)} \end{array}\right.$$

The $\beta_{i}^{HTN}$ and $\beta_{i}^{T2D}$ values were derived from the HTN and T2D GWAS summary statistics in the TPMI cohort. For each pleiotropic ${SNP}_{i}$, we let $\beta_{i}^{HTN}$ and $\beta_{i}^{T2D}$ denote its effect size (log-odds) on HTN and T2D, respectively. This weighting scheme ensures that the coupled PRSs reflect the cumulative burden of variants that synergistically increase (or decrease) risk for both diseases, whereas the uncoupling PRSs capture the net imbalance in genetic effects between the two traits, minimizing the cancelation of effects in opposing directions. To facilitate comparison of effect sizes across different genetic architectures, all PRSs were standardized to a mean of 0 and a standard deviation of 1 prior to downstream analyses.

### 2.5. Genotyping, Imputation, and Quality Control

Genomic DNA was extracted from blood samples and genotyped using the Illumina Asian Screen Array. Standard quality control (QC) procedures were applied at both the variant and individual levels, including filters for missing call rate (<5%), minor allele frequency (MAF > 1%), and Hardy–Weinberg equilibrium (*p* > 1 × 10^−6^). Qualified samples were phased with SHAPEIT v2 and imputed into the 1000 Genomes Project Phase III reference panel using IMPUTE v2. Post-imputation QC removed variants with MAF < 0.01, missing rate ≥ 5%, or imputation quality score (INFO) < 0.7.

### 2.6. Definition of Covariates

All covariates were derived from baseline assessments in the FISSIC. Age and body mass index (BMI) were treated as continuous variables. Sex, baseline comorbidities (including T2D, HTN, and dyslipidemia), and lifestyle factors (including smoking status, drinking status, and physical activity) were used as categorical variables. For covariates with less than 10% missing data, we used mean imputation for continuous variables and treated missing values as a separate category for categorical variables. More details can be found in Method S2 Supplementary File S1.

### 2.7. Coupling/Uncoupling PRS and Comorbidity of HTN and T2D

To investigate whether distinct modes of genetic pleiotropy influence comorbidity of HTN and T2D development, we examined the association between coupling and uncoupling PRSs and the comorbidity of HTN and T2D in the FISSIC. Individuals were classified into mutually exclusive categories: comorbidity (both HTN and T2D), single disease (HTN or T2D), and no disease (neither HTN nor T2D, serving as the reference group).

To evaluate the associations between polygenic risk scores (PRSs) and disease outcomes, we fitted multivariable logistic regression models, adjusting for age and sex. Given the family-based structure of the validation cohort, observations within the same family may not be independent. To account for potential intra-family correlations and ensure the robustness of our estimates, we calculated cluster-robust standard errors clustered by family ID using the sandwich package in R. The statistical significance of PRSs were assessed using Wald tests based on these robust standard errors. Odds ratios (ORs) and 95% confidence intervals (CIs) were reported. To explore the potential risk stratification, we conducted stratified analyses across the aforementioned covariates. Specifically, we included multiplicative interaction terms in separate models, with age, sex, and specific covariates and their corresponding PRSs retained to estimate the interaction effects.

### 2.8. Cardiovascular Outcomes

Cardiovascular outcomes were estimated in the FISSIC cohort from baseline enrollment (since 2007) to 31 December 2024. Three primary endpoints were evaluated: (1) coronary heart disease (CHD), (2) ischemic stroke (IS), and (3) hemorrhagic stroke (HS). All diagnoses of disease were identified through linkage to national health insurance claim databases and supplemented by hospital discharge records, death certificates, and medical chart reviews. Diagnoses were confirmed based on International Classification of Diseases, 10th Revision (ICD-10) codes: CHD (I20–I25), IS (I63), and HS (I61). Events were adjudicated by trained physicians blinded to participants’ genetic profiles, with validation requiring consistent clinical documentation or imaging reports (for stroke subtypes).

### 2.9. Coupling/Uncoupling PRS and Cardiovascular Outcomes

We examined the association between coupling and uncoupling PRSs and the risk of incident cardiovascular diseases using separate logistic regression models for each of the three outcomes: CHD, IS, and HS. Each PRS was modeled as a continuous predictor representing per one SD increase in genetic burden. All models were adjusted for age and sex. To account for potential intra-family correlations and ensure the robustness of our estimates, we also calculated cluster-robust standard errors clustered by family ID. Subgroup analysis and interaction analysis was performed by baseline covariates. The multiplicative interaction was evaluated by including a product term of the PRS and the subgroup variable in the regression model. Results are presented as ORs with 95% confidence intervals. Results with *p* < 0.05 were considered statistically significant. Exploratorily, we further applied the Benjamini–Hochberg procedure to control the false discovery rates (FDRs) in the subgroup analysis and interaction analysis.

### 2.10. Functional Annotation and Pathway Enrichment Analysis

To gain biological insights into the identified pleiotropic loci, we performed comprehensive functional annotation and pathway enrichment analysis using SNPnexus, a state-of-the-art web-based server for the functional interpretation of human genomic variation [35]. For each SNP from our coupling and uncoupling pleiotropic loci, we submitted its genomic coordinates (GRCh38/hg19 assembly) to the SNPnexus platform (https://www.snp-nexus.org/v4/, accessed on 5 March 2026) (Method S3 Supplementary File S1).

## 3. Results

### 3.1. Coupling and Uncoupling Pleiotropy Loci for Hypertension and Type 2 Diabetes

**Figure 1:**
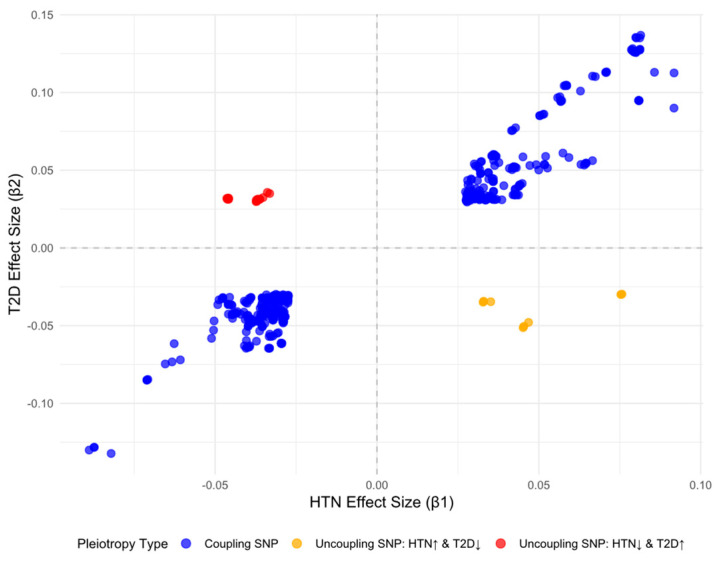
Direction of effect for pleiotropic loci associated with hypertension and type 2 diabetes. Scatter plot showing the effect sizes (β) of 463 pleiotropic SNPs significantly associated with both hypertension (HTN, *x*-axis, β_1_) and type 2 diabetes mellitus (T2D, *y*-axis, β_2_) in the Taiwan Precision Medicine Initiative (TPMI) cohort, identified using a conditional false discovery rate (cFDR) approach (*p* < 0.05). Each point represents a SNP, color-coded by pleiotropy type: blue denotes coupling SNPs (concordant effects on both traits); orange indicates uncoupling SNPs with HTN-increasing but T2D-decreasing effects; red indicates uncoupling SNPs with HTN-decreasing but T2D-increasing effects. Dashed lines mark null effects (β = 0) for each trait. SNPs in the first and third quadrants (same direction of effects) are classified as coupling loci (*n* = 439), reflecting shared genetic risk or protection. SNPs in the second and fourth quadrants (opposite effects) are defined as uncoupling loci (*n* = 24), indicating antagonistic pleiotropy.

Genetic correlation analysis revealed a substantial genome-wide genetic correlation between HTN and T2D with r_g_ of 0.47. A total of 463 loci was significantly associated with both traits at a conjunctional FDR threshold of <0.05 in the TPMI (Table S1). As illustrated in Figure 1, these loci were clearly classified as 439 coupling loci, whose risk alleles exert concordant effects on both HTN and T2D, and 24 uncoupling loci (including 16 T2D increasing—HTN decreasing loci, and 8 HTN increasing–T2D decreasing loci), whose risk alleles have opposing effects on the two diseases. Notably, the coupling loci demonstrated strong evidence of colocalization between HTN and T2D (PP.H4 = 99%) (Table S2). PRS for coupling and uncoupling effects were subsequently constructed using the lead SNPs from the respective loci.

### 3.2. Association of Coupling and Uncoupling Pleiotropy Loci with Comorbidity in FISSIC

A total of 1205 participants from 536 families were included in FISSIC. Family structures are shown in Table S3. The mean age of participants was 58.4 years (SD = 10.7), with females comprising 42.7% of the cohort; notably, 22.2% (*n* = 268) had comorbidity of hypertension and type 2 diabetes (Table 1). The baseline characteristics of participants stratified by the median of coupling and uncoupling PRSs are presented in Tables S4–S6.

After adjusting for age, sex, and family-clustering effects, the standardized coupling PRS modeled as a continuous variable was significantly associated with single T2D (OR = 1.53 per one standard deviation increase, 95% CI: 1.08–2.18; *p* = 0.017) but not significantly associated with HTN (OR = 1.13; 95% CI: 0.96–1.34; *p* = 0.150) or comorbidity (OR = 1.15, 95% CI: 0.95–1.38; *p* = 0.156). Moreover, the association of standardized HTN-risk-increasing uncoupling PRS with HTN risk (OR = 1.10; 95% CI: 0.92–1.30, *p* = 0.296) and the association of standardized T2D-risk-increasing uncoupling PRS with T2D risk (OR = 1.28; 95% CI: 0.93–1.77, *p* = 0.133) did not reached significance. (Table 2). Subgroup analysis found that the T2D-risk-increasing uncoupling PRS was associated with an increased risk of comorbidity in participants aged 60 years or older. (Tables S7–S9).

### 3.3. Association of Coupling and Uncoupling PRSs with Cardiovascular Outcomes in FISSIC

As shown in Table 3, individuals with HTN-T2D comorbidity exhibited markedly elevated prevalence and substantially increased odds of cardiovascular outcomes compared to those with neither condition.

Table 4 presents associations between three PRS types and major cardiovascular events. The coupling PRS and both uncoupling PRSs showed no significant associations with higher risks of CHD, IS, or HS (*p* > 0.05 for each).

Subgroup analyses revealed significant heterogeneity in the associations between PRSs and cardiovascular outcomes across several stratification factors (Table 5 and Table S10). Given the exploratory nature and limited sample size in certain subgroups, results should be interpreted with caution. Regarding coupling PRSs, a significant interaction was observed with HDL-C levels in relation to CHD risk (*p* = 0.024). Stratified analysis indicated a differential association between individuals with low HDL-C levels (OR = 0.89, 95% CI: 0.75–1.05) and those with high HDL-C levels (OR = 1.04, 95% CI: 0.88–1.23). For HTN-risk-increasing uncoupling PRSs, significant interactions were identified with multiple metabolic and lifestyle factors concerning the risk of HS (although the point estimate suggested a potential association, the limited number of hemorrhagic stroke events preclude definitive conclusions). Specifically, we observed significant interactions with baseline dyslipidemia (*p* _interaction_ = 0.003), baseline hypertension (*p* _interaction_ = 0.004), LDL-C levels (*p* _interaction_ = 0.005), TC levels (*p* _interaction_ = 0.017), and TG levels (*p* _interaction_ = 0.027). Additionally, the association between HTN-risk-increasing uncoupling PRSs and HS risk varied significantly by baseline T2D status (*p* _interaction_ = 0.021) and sports participation (*p* _interaction_ = 0.042). Specifically, among individuals with baseline dyslipidemia, the HTN-risk-increasing uncoupling PRS was associated with a significantly elevated risk of HS (OR = 1.65; 95% CI: 1.05–2.58; *p* = 0.028); conversely, in those without baseline hypertension, it was associated with a significantly reduced risk of HS (OR = 0.30; 95% CI: 0.13–0.71; *p* = 0.006). Furthermore, in physically active individuals, this PRS was associated with a higher risk of IS (OR = 2.60; 95% CI: 1.04–6.47; *p* = 0.041). Finally, for T2D-risk-increasing uncoupling PRSs, a significant interaction was detected with smoking status in relation to IS risk (*p* _interaction_ = 0.044), suggesting that the genetic risk association differs between smokers and non-smokers. After FDR correction, no interactions remained statistically significant (FDR-adjusted *p* < 0.05).

### 3.4. Pathway Enrichment, Functional Annotation, and Phenotypic Associations

To gain deeper insights into the underlying mechanisms of the two types of pleiotropic loci, we first mapped the identified SNP loci to their putative target genes (Tables S11 and S12). A total of 439 coupling SNPs were primarily located within or near genes of high biological relevance, including FTO, BDNF, WT1, and KLF14. In contrast, 24 uncoupling SNPs mapped to a distinct set of genes, notably HNF4A, DOT1L and WNT2B. This differential gene mapping suggests that coupling and uncoupling pleiotropy may operate through divergent molecular pathways.

We further explored the functional characteristics of these pleiotropic variants. As shown in Tables S13 and S14, coupling SNPs were significantly enriched in pathways related to hemostasis, immune activation, intracellular signaling, and neurodevelopmental regulation. In stark contrast, uncoupling SNPs exhibited enrichment primarily in developmental biology, transcriptional regulation, and WNT signaling. Key pathways included regulation of beta cell development (*p* = 0.016), signaling by WNT (*p* = 0.117) and WNT ligand biogenesis and trafficking (*p* = 0.010), implicating pancreatic islet development and morphogenesis.

As shown in Tables S15 and S16, phenome-wide association analysis of the 439 coupling SNPs revealed a striking enrichment for obesity and related anthropometric traits. Among the most significantly associated phenotypes were BMI (e.g., rs6265, *p* = 7 × 10^−89^; rs1421085, *p* = 1 × 10^−300^), waist–hip ratio (e.g., rs6265, *p* = 4 × 10^−17^), hip circumference (e.g., rs6265, *p* = 1 × 10^−14^), and obesity across multiple classes (e.g., rs2030323, *p* = 3 × 10^−22^). These associations were consistently observed across diverse ancestries, including European, East Asian, and multi-ethnic cohorts. In stark contrast, uncoupling SNPs showed no significant links to adiposity measures.

## 4. Discussion

In this two-stage study, we leveraged two independent cohorts to investigate how the shared genetic architecture of HTN and T2D—differentiated between coupling and uncoupling pleiotropy—are associated with cardiovascular disease outcomes. Using the largest available GWAS dataset from East Asian populations for both traits, we identified 463 loci significantly associated with both HTN and T2D, including 439 coupling loci and 24 uncoupling loci. In the second stage, we constructed separate PRSs based on coupling and uncoupling loci, and evaluated their associations with cardiovascular outcomes in an independent family-based cohort. Overall, although the coupling and uncoupling forms of pleiotropy were not directly significantly associated with cardiovascular outcomes, in exploratory analysis, they exhibited distinct gene–environment interactions across different cardiovascular risk profiles. The association between the coupling PRS and CHD was significantly modified by HDL-C levels. In contrast, the HTN-risk-increasing uncoupling PRS exhibited extensive interactions with baseline metabolic comorbidities, lipid profiles, and lifestyle factors, thereby significantly modifying its impact on the risks of CHD, IS, and HS. Specifically, among individuals with baseline dyslipidemia, the HTN-risk-increasing uncoupling PRS was associated with a significantly elevated risk of HS; conversely, in those without baseline hypertension, it was associated with a significantly reduced risk of HS. Furthermore, in physically active individuals, this PRS was associated with a higher risk of IS. Additionally, an interaction was observed between the T2D-risk-increasing uncoupling PRS and smoking status regarding their association with IS. A positive correlation was noted among smokers, although this association did not reach statistical significance. This study provides the largest-scale evidence from East Asian populations on pleiotropy of HTN-T2D and offers novel insights into potential cardiovascular disease prevention, leveraging both coupling and uncoupling forms of pleiotropy.

Although HTN and T2D frequently co-occur clinically [9,10], the detailed genetic pleiotropy between them and its contribution to cardiovascular risk remain poorly understood. Recent multivariate genomic studies have reported a genetic correlation exceeding 40% between HTN and T2D [20,21], which aligns well with epidemiological observations in real-world populations [9,10]. Our research results further support that HTN and T2D share a common genetic risk, namely horizontal pleiotropy, rather than simple vertical pleiotropy mentioned in prior studies [36,37]. Among the 463 pleiotropic loci identified, we classified these based on the concordance of their effect directions on the two traits: 439 were coupling loci and 24 were uncoupling loci. While this classification strategy has been applied to continuous traits [22], we extended its use to dissect the directionality of locus effects across distinct disease phenotypes. Notably, the concept of uncoupling pleiotropy is similar to antagonistic pleiotropy—a term traditionally used to describe genetic variants that confer opposing fitness effects or biological actions across different environmental contexts or life stages [25,38]. In this study, the uncoupling pleiotropy referred to specifically denotes SNPs that exert opposing effects on two distinct traits, as opposed to coupling pleiotropy, where the same allele influences both traits in the same direction. Notably, the greater number of coupling loci compared to uncoupling loci is biologically consistent with the extensive shared genetic architecture between HTN and T2D [20,21]. The high comorbidity of these conditions is largely driven by shared pathophysiological pathways—such as insulin resistance and inflammation—that concurrently increase susceptibility to both diseases. Consequently, alleles exerting concordant effects are naturally more prevalent, whereas variants that increase risk for one condition while protecting against the other are expected to be rare due to these overlapping biological mechanisms. Our findings further suggest that coupling and uncoupling loci differ in both predictive utility for clinical outcomes and biological mechanisms. This distinction offers an insightful framework for future pleiotropy studies.

In this study, we observed that coupling and uncoupling PRSs aligned well with HTN and T2D risk architectures: the coupling PRS was associated with increased risk for T2D, whereas the other associations were not significant although the effect estimates were directionally consistent with our hypothesis. These findings strongly support the validity of our PRS constructions. While a positive association was observed between the coupled PRS and HTN/T2D comorbidity, it was not statistically significant.

The exploratory interaction analysis provides some insights into potential precision prevention and control. Although the initial analyses did not reveal significant associations between the pleiotropic PRSs and cardiovascular events, subgroup analyses uncovered distinct patterns of association across patients with varying baseline characteristics. Specifically, the association between the coupling PRS (which is closely linked to HTN and T2D) and CHD was inconsistent across patients with different HDL-C levels. The HTN-risk-increasing uncoupling PRS was associated with HS—a finding consistent with the established role of hypertension as the primary risk factor for HS [39]. Furthermore, this association exhibited extensive interactions with multiple lipid parameters, including LDL-C, TC, and TG. Among individuals with elevated LDL-C or TC, the HTN-risk-increasing uncoupling PRS was associated with a significantly higher risk of HS. Our findings suggest that the HTN-risk-increasing uncoupling PRS (genetic risk of hypertension, particularly the component that may lower T2D risk) might synergistically promote the development of HS with lipid metabolism. It is important to note that our findings did not survive multiple testing corrections. Given the exploratory nature of this study, these results underscore the need for future research with larger sample sizes.

Exploration at the mechanistic level shows that the coupling loci were strongly associated with BMI, which is highly consistent with our findings and previous studies [23,40]. These coupling loci predominantly map to or overlap key genes, including FTO, BDNF, and WT1, and are located upstream of KLF14. Moreover, we observed that a substantial proportion of these loci reside within non-coding RNA regions, particularly long intergenic non-coding RNAs (lincRNAs) and antisense RNAs, suggesting that their effects are likely mediated through regulatory mechanisms rather than direct alterations of protein-coding sequences. Meanwhile, only a small fraction (5.2%) was classified as uncoupling loci overall, a proportion consistent with a recent report [23]. These uncoupling loci overlap genes such as HNF4A, and DOT1L, and are associated with beta cell dysfunction [41] and pulse pressure-related traits [42]. This provides biological plausibility for the uncoupling concept and clearly distinguishes it from the obesity-centered mechanisms underlying coupling loci.

Our study innovatively deconstructs genetic pleiotropy by systematically classifying the shared loci between HTN and T2D into two functionally distinct subtypes—coupling and uncoupling. This framework provides a novel conceptual lens for understanding the heterogeneous genetic underpinnings of comorbidity. Furthermore, we address a critical gap in the literature by focusing on East Asian populations. The two-stage design ensures that our findings are both population-representative and biologically credible at the individual level. Critically, our findings carry direct implications for precision prevention. We acknowledge our study have several limitations. First, the discovery stage relied on publicly available GWAS summary statistics, which inherently cannot account for individual-level confounders. Moreover, this approach may miss rare variants (MAF < 1%) [43] and higher-order genetic interactions. Second, while the cFDR method enhances sensitivity for detecting pleiotropic signals, it may still introduce false positives, particularly when the auxiliary trait exhibits only weak association signals. Third, our functional interpretations of implicated genes are primarily based on bioinformatic annotations and lack direct experimental validation. Thus, these mechanistic hypotheses require confirmation through functional studies. Fourth, although several nominal interactions showed low unadjusted *p*-values, these did not survive rigorous correction for multiple testing. These findings should be considered hypothesis-generating and warrant validation in future larger-scale studies. Fifth, the potential for unassessed residual confounding cannot be entirely ruled out. Sixth, although our family-based cohort offers rich phenotyping, approximately 10 years of follow-up, and reduced population stratification, the number of endpoints, such as myocardial infarction and hemorrhagic stroke, remains relatively limited. Furthermore, compared to the discovery population used in the first stage, the sample size of this family-based validation cohort is relatively smaller. This constrains statistical power and results in wider confidence intervals for some effect estimates. Specifically, the number of hemorrhagic stroke events was relatively small, which limits the statistical power to detect significant associations or interactions within this specific subgroup. Consequently, the findings regarding hemorrhagic stroke should be considered hypothesis-generating. Future validation in larger prospective cohorts or through meta-analyses will be essential to confirm these associations or interactions. Finally, caution is warranted when generalizing the findings of this study to populations beyond East Asia. Since coupling/uncoupling PRSs are novel metrics without extensive prior validation, these scores are intended as exploratory metrics to quantify specific modes of pleiotropy rather than as definitive clinical predictors.

## 5. Conclusions

By dissecting the shared genetic architecture of hypertension and type 2 diabetes into coupling and uncoupling pleiotropy, this study suggests that these forms are potentially associated with different risks for specific cardiovascular events, especially in specific baseline conditions. While these findings are exploratory and warrant validation in larger cohorts, our study proposes a framework for leveraging directional pleiotropy to refine risk stratification. This approach may ultimately help prioritize high-risk subgroups and inform precision prevention strategies in East Asian.

## Figures and Tables

**Table 1:** Characteristics of participants in FISSIC.

Characteristics	Mean/Freq	SD/Percent
Age (year), mean (SD)	58.4	10.7
Female, N (%)	515	42.7
Education level, N (%)		
Less than primary school	221	18.6
Primary school	286	24.0
Junior high school	497	41.8
Senior high school or vocational secondary	150	12.6
Associate degree or higher	36	3.0
Marriage, N (%)	1079	91.0
Smoking, N (%)	633	52.5
Drinking, N (%)	498	42.0
Sports, N (%)	38	3.2
BMI (kg/m^2^), mean (SD)	26.1	3.63
TC (mmol/L), mean (SD)	3.1	0.9
TG (mmol/L), mean (SD)	1.5	1.2
HDL-C (mmol/L), mean (SD)	0.9	0.3
LDL-C (mmol/L), mean (SD)	2.3	0.8
Hypertension, N (%)	778	64.6
Type 2 diabetes, N (%)	333	27.6
Dyslipidemia, N (%)	378	31.4
Comorbidity of HTN and T2D, N (%)	268	22.2
Coupling PRS	−2.6 × 10^−4^	4.5 × 10^−3^
HTN-risk-increasing uncoupling PRS	2.9 × 10^−2^	1.6 × 10^−2^
T2D-risk-increasing uncoupling PRS	2.1 × 10^−2^	3.2 × 10^−2^

Abbreviations: SD, standard difference; HDL-C, high-density lipoprotein cholesterol; LDL-C, low-density lipoprotein cholesterol; TC, total cholesterol; TG, triglycerides; HTN, hypertension; T2D, type 2 diabetes.

**Table 2:** Association of coupling and uncoupling PRSs with the comorbidity of HTN and T2D.

Exposure	Outcome	OR (95% CI) ^1^	*p*
Coupling PRS	Comorbidity	1.15 (0.95, 1.38)	0.156
	HTN only	1.13 (0.96, 1.34)	0.150
	T2D only	1.53 (1.08, 2.18)	0.017
HTN-risk-increasing uncoupling PRS	Comorbidity	1.03 (0.85, 1.24)	0.797
	HTN only	1.10 (0.92, 1.30)	0.296
	T2D only	1.03 (0.71, 1.47)	0.893
T2D-risk-increasing uncoupling PRS	Comorbidity	1.15 (0.96, 1.37)	0.121
	HTN only	1.07 (0.89, 1.29)	0.461
	T2D only	1.28 (0.93, 1.77)	0.133

Abbreviations: PRS, polygenic risk score; HTN, hypertension; T2D, type 2 diabetes; OR, odds ratio. Odds ratios represent the change in odds per one standard deviation increase in the PRS. ^1^ The reference group consisted of all study participants who have neither HTN nor T2D.

**Table 3:** Prevalence and odds ratios for cardiovascular diseases by HTN-T2D comorbidity status.

Disease	Group	Case	N	Prevalence, %	OR (95% CI) ^1^	*p*
CHD	Comorbidity	369	506	72.92	35.14 (19.23, 64.21)	<0.001
CHD	Single disease	229	501	45.71	10.35 (5.89, 18.16)	<0.001
CHD	Neither	17	198	8.59	Ref	Ref
IS	Comorbidity	252	506	49.80	30.51 (13.65, 68.19)	<0.001
IS	Single disease	135	501	26.95	10.52 (4.75, 23.29)	<0.001
IS	Neither	7	198	3.54	Ref	Ref
HS	Comorbidity	9	506	1.78	-	-
HS	Single disease	6	501	1.20	-	-
HS	Neither	0	198	0.00	Ref	Ref

Abbreviations: HTN, hypertension; T2D, type 2 diabetes; OR, odds ratio; CI, confidence interval; CHD, coronary heart disease; IS, ischemic stroke; HS, hemorrhagic stroke. ^1^ Odds ratios were estimated from logistic regression models adjusted for age, sex, and family clustering (using generalized estimating equations or robust standard errors to account for relatedness).

**Table 4:** Association of coupling and uncoupling PRSs with cardiovascular outcomes.

PRS Type	Outcome	OR (95% CI)	*p*
Coupling PRS	CHD	0.97 (0.86, 1.09)	0.588
	IS	0.96 (0.85, 1.08)	0.515
	HS	1.15 (0.88, 1.52)	0.306
HTN-risk-increasing uncoupling PRS	CHD	1.05 (0.93, 1.18)	0.473
	IS	1.03 (0.92, 1.17)	0.590
	HS	1.01 (0.77, 1.32)	0.964
T2D-risk-increasing uncoupling PRS	CHD	1.06 (0.94, 1.20)	0.326
	IS	1.01 (0.90, 1.14)	0.853
	HS	0.86 (0.65, 1.15)	0.317

Abbreviations: PRS, polygenic risk score; OR, odds ratio; CI, confidence interval; CHD, coronary heart disease; IS, ischemic stroke; HS, hemorrhagic stroke; HTN, hypertension; T2D, type 2 diabetes.

**Table 5:** Subgroup analyses of coupling and uncoupling PRSs and cardiovascular events—results with significant interaction.

Exposure	Outcome	Subgroup Variable	Subgroup Level	OR (95% CI)	*p*	*p* _interaction_
Coupling PRS	CHD	HDL-C	Low	0.89 (0.75, 1.05)	0.170	0.024
Coupling PRS	CHD	HDL-C	High	1.04 (0.88, 1.23)	0.664	0.024
HTN-risk-increasing uncoupling PRS	HS	Baseline Dyslipidemia	Yes	1.65 (1.05, 2.58)	0.028	0.003
HTN-risk-increasing uncoupling PRS	HS	Baseline Dyslipidemia	No	0.72 (0.51, 1.04)	0.077	0.003
HTN-risk-increasing uncoupling PRS	HS	Baseline HTN	Yes	1.18 (0.89, 1.58)	0.249	0.004
HTN-risk-increasing uncoupling PRS	HS	Baseline HTN	No	0.30 (0.13, 0.71)	0.006	0.004
HTN-risk-increasing uncoupling PRS	HS	LDL-C	High	1.53 (0.98, 2.40)	0.065	0.005
HTN-risk-increasing uncoupling PRS	HS	LDL-C	Low	0.80 (0.56, 1.14)	0.209	0.005
HTN-risk-increasing uncoupling PRS	HS	TC	High	1.46 (0.90, 2.36)	0.123	0.017
HTN-risk-increasing uncoupling PRS	HS	TC	Low	0.84 (0.60, 1.17)	0.300	0.017
HTN-risk-increasing uncoupling PRS	HS	TG	Low	0.92 (0.64, 1.33)	0.661	0.027
HTN-risk-increasing uncoupling PRS	HS	TG	High	1.09 (0.71, 1.68)	0.701	0.027
HTN-risk-increasing uncoupling PRS	CHD	Baseline T2D	Yes	0.83 (0.65, 1.06)	0.129	0.021
HTN-risk-increasing uncoupling PRS	CHD	Baseline T2D	No	1.13 (0.99, 1.30)	0.067	0.021
HTN-risk-increasing uncoupling PRS	IS	Sports	Yes	2.60 (1.04, 6.47)	0.041	0.042
HTN-risk-increasing uncoupling PRS	IS	Sports	No	1.01 (0.89, 1.14)	0.909	0.042
T2D-risk-increasing uncoupling PRS	IS	Smoking	Yes	1.15 (0.97, 1.37)	0.103	0.044
T2D-risk-increasing uncoupling PRS	IS	Smoking	No	0.90 (0.76, 1.06)	0.195	0.044

Abbreviations: PRS, polygenic risk score; OR, odds ratio; CI, confidence interval; CHD, coronary heart disease; IS, ischemic stroke; HS, hemorrhagic stroke; HTN, hypertension; T2D, type 2 diabetes; HDL-C, high-density lipoprotein cholesterol; LDL-C, low-density lipoprotein cholesterol; TC, total cholesterol; TG, triglycerides.

## Data Availability

Data are not publicly available due to restrictions.

## Supplementary Materials

The following supporting information can be downloaded at: https://www.mdpi.com/article/10.3390/biomedicines14061404/s1. Method S1 File S1. Genetic correlation analysis and colocalization analysis. Method S2 File S1. Definition of covariates. Method S3 File S1. Functional Annotation and Pathway Enrichment Analysis. Table S1. Coupling and uncoupling SNPs and their directions of effect. Table S2. Colocalization analysis of coupling and uncoupling SNPs. Table S3. Family structure distribution. Table S4. Characteristics of participants in FISSIC classified by the median of the coupling PRSs. Table S5. Characteristics of participants in FISSIC classified by the median of the HTN-risk-increasing uncoupling PRSs. Table S6. Characteristics of participants in FISSIC classified by the median of the T2D-risk-increasing uncoupling PRSs. Table S7. Association of coupling PRSs with comorbidity of HTN and T2D. Table S8. Association of HTN-risk-increasing uncoupling PRSs with comorbidity of HTN and T2D. Table S9. Association of T2D-risk-increasing uncoupling PRSs with comorbidity of HTN and T2D. Table S10. Subgroup analyses of coupling and uncoupling PRSs and cardiovascular events. Table S11. Mapping of coupling SNPs to genes. Table S12. Mapping of uncoupling SNPs to genes. Table S13. Pathway enrichment of coupling pleiotropic loci. Table S14. Pathway enrichment of uncoupling pleiotropic loci. Table S15. Phenotypic associations of coupling SNPs. Table S16. Phenotypic associations of uncoupling SNPs.

## Abbreviations

The following abbreviations are used in this manuscript:

HTN	Hypertension
T2D	Type 2 Diabetes
CVD	Cardiovascular Disease
CHD	Coronary Heart Disease
IS	Ischemic Stroke
HS	Hemorrhagic Stroke
PRS	Polygenic Risk Score
GWAS	Genome-Wide Association Study
cFDR	Conjunctional False Discovery Rate
LD	Linkage Disequilibrium
SNP	Single Nucleotide Polymorphism
HDL-C	High-Density Lipoprotein Cholesterol
LDL-C	Low-Density Lipoprotein Cholesterol
TC	Total Cholesterol
TG	Triglycerides
BMI	Body Mass Index
MAF	Minor Allele Frequency
TPMI	Taiwan Precision Medicine Initiative
FISSIC	Fangshan Family-Based Ischemic Stroke Study in China
COLOC	Colocalization Analysis
