# Supplementary material for: Coupling and Uncoupling Pleiotropy Between Hypertension and Type 2 Diabetes Contribute to Exploring Potential Heterogeneity in Cardiovascular Risk in East Asian Population
Source: Biomedicines. 2026 Jun 22;14(6):1404. doi: 10.3390/biomedicines14061404 (PMC13297183; doi:10.3390/biomedicines14061404)
Supplement: Supplementary file 1 [file biomedicines-14-01404-s001.zip › 20260620-Supplementary File S1.pdf]

Supplementary File S1

Method S1

Genetic correlation analysis and colocalization analysis

To quantify the overall shared genetic architecture between HTN and T2D, we estimated their genome-wide genetic correlation (r_g) using MTAG[32]. This analysis was performed on the GWAS summary statistics from the TPMI cohort, following standard protocols to account for sample overlap and linkage disequilibrium (LD) structure.

To investigate whether the observed genetic correlation at specific genomic loci was driven by shared causal variants, we conducted colocalization analysis using the COLOC method. COLOC uses approximate Bayes factors derived from GWAS summary statistics to compute posterior probabilities for five mutually exclusive hypotheses:

(H_0) no association with either trait in the region;

(H_1) association with HTN only;

(H_2) association with T2D only;

(H_3) association with both traits but driven by two distinct causal variants;

(H_4) association with both traits driven by a single shared causal variant.

We applied COLOC to the TPMI GWAS summary statistics for HTN and T2D within a ±500 kb window centered on each lead pleiotropic SNP. Following standard practice, we used the default priors: $P_1 = 1 \times 10^{-4}$ (prior probability of a SNP being associated with HTN), $P_2 = 1 \times 10^{-4}$ (prior probability of a SNP being associated with T2D), and $P_{12} = 1 \times 10^{-5}$ (prior probability of a SNP being causally associated with both traits). We considered a posterior probability for hypothesis H_4 (PPH_4) ≥ 0.8 as strong evidence of colocalization, indicating that the same underlying causal variant likely influences both HTN and T2D at that locus[33,34].

Method S2

Definition of covariates

All covariates were derived from baseline assessments in FISSIC. Age and BMI were included as continuous variables and standardized to represent per–standard deviation effects. Sex was modeled as a binary variable. Baseline comorbidities, including T2D, HTN, and dyslipidemia, were defined as binary indicators based on prevalent status at enrollment. Specifically, baseline T2D was defined by physician diagnosis, use of glucose-lowering medications, fasting blood glucose ≥7.0 mmol/L, or 2-hour post-oral glucose tolerance test glucose ≥11.1 mmol/L; HTN followed the 1999 WHO-ISH criteria (systolic blood pressure ≥140 mmHg, diastolic blood pressure ≥90 mmHg, or antihypertensive medication use); and dyslipidemia was defined by elevated total cholesterol (≥6.2 mmol/L), LDL-C (≥4.1 mmol/L), triglycerides (≥2.3 mmol/L), low HDL-C (<1.0 mmol/L in men or <1.3 mmol/L in women), or current lipid-lowering therapy. Lifestyle factors included smoking status (dichotomized as “current” versus “never/former”), alcohol consumption (“current” versus “never/former”), and physical activity (defined as engaging in moderate- or vigorous-intensity physical activity at least once per week). Body mass index (BMI, kg/m^2) was calculated from body measurement at baseline. For covariates with less than 10% missing data, we used mean imputation for continuous variables and treated missing values as a separate category for categorical variables. To account for the familial correlation inherent in the FISSIC design, we employed family-specific random intercepts and cluster-robust standard errors based on family ID in logistic models, ensuring appropriate adjustment for within-family dependence across all analyses.

Method S3

Functional Annotation and Pathway Enrichment Analysis

To gain biological insights into the identified pleiotropic loci, we performed comprehensive functional annotation and pathway enrichment analysis using SNPnexus, a state-of-the-art web-based server for the functional interpretation of human genomic variation[35]. For each SNP from our coupling and uncoupling pleiotropic loci, we submitted its genomic coordinates (GRCh38/hg19 assembly) to the SNPnexus platform (https://www.snp-nexus.org/v4/). The tool was used to annotate these variants with a wide range of functional information, including their mapping to known genes (using multiple gene annotation systems: Ensembl, RefSeq, UCSC, CCDS), predicted consequences on protein coding (e.g., missense, synonymous), regulatory elements (e.g., ENCODE, Roadmap Epigenomics), and crucially, their association with known phenotypes from curated databases such as COSMIC and ClinVar.

Furthermore, to identify the broader biological processes and pathways potentially perturbed by our gene sets, we utilized SNPnexus's integrated Reactome pathway enrichment analysis module. This feature maps the genes harboring our lead SNPs to their associated Reactome pathways and performs a Fisher's Exact test to determine if any pathways are statistically over-represented within our gene list compared to the background universe of all genes in Reactome. The resulting enriched pathways provide a systems-level view of the molecular mechanisms underlying the shared genetic architecture of HTN and T2D.
